# Low-Dose Paracetamol Treatment Protects Neuronal Oxidative Stress and Neuroinflammation in D-Galactose-Induced Accelerated Aging Model

**DOI:** 10.1155/sci5/5559483

**Published:** 2025-09-18

**Authors:** Chuchard Punsawad, Paweena Kaewman, Tachpon Techarang, Diana Sketriene, Laddawan Lalert

**Affiliations:** ^1^Department of Medical Science, School of Medicine, Walailak University, Nakhon Si Thammarat 80160, Thailand; ^2^Center of Excellence in Tropical Pathobiology, Walailak University, Nakhon Si Thammarat 80160, Thailand; ^3^Department of Anatomy, Faculty of Medical Science, Naresuan University, Phitsanulok 65000, Thailand; ^4^Center of Excellence in Medical Biotechnology, Naresuan University, Phitsanulok 65000, Thailand; ^5^Department of Tropical Pathology, Faculty of Tropical Medicine, Mahidol University, Bangkok 10400, Thailand; ^6^Department of Biochemistry and Pharmacology, Medicine, Dentistry and Health Sciences, The University of Melbourne, Victoria 3010, Australia; ^7^Department of Physiology, Faculty of Medical Science, Naresuan University, Phitsanulok 65000, Thailand

## Abstract

Aging increases the risk of neurodegenerative diseases such as Parkinson's and Alzheimer's (PD and AD) which are potentially linked to increased oxidative stress and inflammation. Paracetamol (APAP) is known for its antioxidant and anti-inflammatory properties; however, its potential neuroprotective effects against age-related oxidative stress and neuroinflammation remain inadequately investigated. Therefore, we aimed to examine whether low-dose APAP could mitigate oxidative stress and neuroinflammation in a D-galactose (D-gal)-induced aging model. In our study, fifty adult male ICR mice were divided into five groups (*n* = 10). Except for the normal control group, all mice received D-gal subcutaneous injections (200 mg/kg) and were fed vehicle, 15 or 50 mg/kg APAP, or 100 mg/kg vitamin E daily for six weeks. After treatment, liver function was assessed by serum liver enzyme analysis. The liver and brain pathologies were examined using hematoxylin and eosin staining. Brain oxidative stress was evaluated through malondialdehyde (MDA) measurement. Additionally, immunohistochemistry was used to determine levels of inflammatory cytokines (TNF-α, IL-1β, TGF-β, and IL-10) and the oxidative stress marker, NADPH Oxidase 4 (NOX4). The study found no significant changes in serum liver enzymes or liver morphology among the experimental groups. However, the D-gal group exhibited increased neuronal cell loss, along with elevated levels of MDA and NOX4 in the frontal cortex and hippocampus. Moreover, D-gal mice showed elevated levels of TNF-α, IL-1β, and TGF-β, accompanied by decreased IL-10 levels. Notably, treatment with low-dose APAP and vitamin E mitigated neuronal cell loss, decreased MDA levels, and attenuated NOX4 expression induced by D-gal injection. Furthermore, low-dose APAP, particularly at 50 mg/kg, and vitamin E reversed the alterations in TNF-α, IL-1β, and IL-10 induced by D-gal, while TGF-β was unaffected. We suggest that low-dose APAP exerts antioxidant and anti-inflammatory activities to protect against neurodegeneration in a mouse model of brain aging induced by chronic D-gal injection.

## 1. Introduction

Aging population has been gradually rising worldwide and is estimated to reach 2.1 billion by 2050 [1]. The natural process of aging is involved in progressive structural and functional decline which can develop serious diseases associated with advanced aging such as cardiovascular conditions and neurological disorders [2, 3]. Advanced aging has also been linked to increase risk for developing Alzheimer's disease (AD), Parkinson's disease (PD), and vascular dementia [4]. Numerous theories have been proposed to explain the complex and diverse changes that occur throughout this process, ranging from molecular mechanisms to effects at the organismal level. Among these, increased oxidative stress is widely recognized as a contributing factor to aging, causing damage to cells, tissues, and organs [4]. Excessive production of reactive oxygen species (ROS) during aging can impair cellular components including proteins, lipids, and DNA, which can further contribute to the deterioration of cellular structure and function, ultimately resulting in cell injury and loss [5].

In the brain, elevated oxidative stress is closely linked to the downregulation of neurotrophins and synaptic dysfunction, both of which are critical factors associated with cognitive decline [6, 7]. Additionally, increased oxidative stress can alter the levels of various inflammatory cytokines, thereby promoting an inflammatory response [5]. During aging, fluctuations in anti-inflammatory cytokines such as interleukin-10 (IL-10) and transforming growth factor-β (TGF-β), along with the upregulation of proinflammatory cytokines such as tumor necrosis factor-α (TNF-α) and interleukin-1β (IL-1β), have been reported in several brain regions, including the hippocampus, cortex, amygdala, and thalamus [5]. These cytokine alterations, in combination with elevated oxidative stress, are strongly implicated in the development and progression of neurodegenerative diseases such as AD and PD [8]. As a result, growing attention is being directed toward agents with antioxidants and anti-inflammatory properties as a strategic approach to mitigate the detrimental effects of aging and improve the quality of life in older adults.

D-galactose (D-gal) is a monosaccharide found inside the body and in several natural products, including yogurt, cheese, milk, and butter, in various fruits, such as kiwis, plums, chestnuts, and cherries, and in herbs and vegetables [9]. At normal levels of consumption, D-gal can be entirely metabolized in animals. Conversely, in case of overconsumption, D-gal cannot be completely processed and the accumulation of galactitol in cells can occur. As a result, the brain experiences an excess of free radicals and ROS and an increase in oxidative stress [10]. Indeed, D-gal-induced rodents have proven to be an effective model for studying aging-related brain and neurobehavioral changes [9, 11, 12] and have also been widely used to investigate the potential therapeutic effects of various neuroprotective agents, including both natural and synthetic compounds [11, 13, 14].

Paracetamol (acetaminophen and APAP) is an over-the-counter medication widely used to manage pain and fever. Apart from its antipyloric and antinociceptive properties, APAP is also recognized for its ability to influence neurobehavioral outcomes. In a double-blind randomized controlled study, APAP treatment could enhance cognitive reflection and spatial memory in healthy volunteers [15]. In animal models, APAP treatment has demonstrated nootropic and neuroprotective effects, prospective due to its antioxidant and anti-inflammatory properties. At 15.1 mg/kg, APAP improved colchicine-induced spatial memory loss in rats [16], while higher doses 75–100 mg/kg alleviated memory deficits in models of lipopolysaccharide-induced neuroinflammation [17] and postoperative cognitive decline by modulating hippocampal cytokines [18]. Additionally, our earlier findings demonstrated that APAP at doses of 15 and 50 mg/kg prevented cognitive decline by improving the performance in the novel object recognition (NOR) and Morris water maze (MWM) tests in D-gal-induced aging mice. This memory enhancement was associated with the restoration of brain-derived neurotrophic factor (BDNF) and its receptor, tyrosine kinase B (TrkB), signaling pathways in the frontal cortex and hippocampus [19]. However, it remains unclear whether the antioxidant and anti-inflammatory properties of low-dose APAP underlie the improved cognitive effects observed in the D-gal-induced aging model.

Therefore, this study investigated antioxidant and anti-inflammatory effects of long-term treatment of the two different low doses of APAP (15 or 50 mg/kg) in the frontal cortex and hippocampus, the key brain areas responsible for cognitive function [20], in the D-gal-induced aging mouse model.

## 2. Materials and Methods

### 2.1. Animals and Treatments

A total of fifty male ICR mice aged 6 weeks (weighing 20–25 g) were acquired from Nomura Siam International Co. Ltd. (Pathum Wan, Bangkok, Thailand). Every mouse was housed in a controlled environment with a 12-h light/dark cycle, at 22 ± 2°C, and at a relative humidity of 55 ± 10%. Standard food and drinking water were accessed *ad libitum* by all mice. In this study, all protocols and procedures for using animals were thoroughly evaluated and approved by the Animal Ethics Committee of Walailak University, Thailand (protocol number; WUACUC-65061).

**Figure 1:**
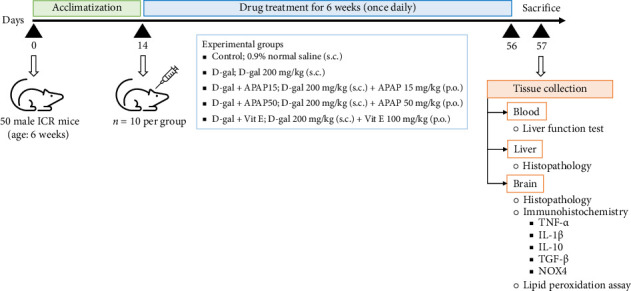
Experimental design. Fifty male ICR mice aged 6 weeks were divided into five groups as follows; control, normal mice orally received with vehicle (0.9% normal saline); D-gal, mice with D-galactose 200 mg/kg injection and orally received with vehicle; D-gal + APAP15, mice with D-galactose injection and orally received with APAP at the dose of 15 mg/kg; D-gal + APAP50, mice with D-galactose injection and orally received with APAP at the dose of 50 mg/kg; D-gal + Vit E, mice with D-galactose injection and orally received with vitamin E at the dose of 100 mg/kg. All treatment was performed for 6 consecutive weeks. TNF-α, tumor necrosis factor-α; IL-1β, Interleukin-1β; IL-10, Interleukin-10; TGF-β, transforming growth factor-β; NOX4, NADPH Oxidase 4.

All mice were acclimated for 14 days and then randomly assigned to five groups. Control group mice (*n* = 10) were subcutaneously (s.c.) injected with the same volume of 0.9% normal saline solution and orally treated with distilled water. D-gal group mice (*n* = 10) were injected (s.c.) with D-gal (Sigma-Aldrich, St. Louis, MO, USA) at a dose of 200 mg/kg and gavaged with distilled water. D-gal + APAP15 and D-gal + APAP50 group mice (*n* = 10) were injected (s.c.) with D-gal at a dose of 200 mg/kg followed by oral gavage with 15 and 50 mg/kg of APAP, respectively (TYLENOL, OLIC (Thailand) Ltd., Ayutthaya, Thailand). D-gal + Vit E group mice (*n* = 10) were injected (s.c.) with D-gal at a dose of 200 mg/kg followed by an oral gavage with active Vit E (α-tocopherol, cat no. 10191-41-0, Merck Millipore, MA, USA) at a dose of 100 mg/kg [21]. The dose and duration of D-gal treatment applied in this study closely matched those established by a previous study [22]. The doses of APAP (15 and 50 mg/kg) employed in this study were selected based on previous findings demonstrating dose-dependent effects of APAP on cognitive function. A study by Ishida et al. reported that a low dose (15.1 mg/kg) enhanced memory performance in the MWM, while higher doses had no effect or were detrimental [23]. In addition, our preliminary data (unpublished) showed that long-term administration of APAP at 50 mg/kg improved both NOR and MWM performance in normal adult rats, suggesting potential nootropic effects. These findings guided our selection of the two low-dose regimens for evaluating neuroprotective effects in the D-gal-induced aging model. Additionally, vitamin E was used as a positive control due to its antioxidant and neuroprotective properties [24]. The treatment was carried out once a day for six weeks with 30-min intervals between D-gal and APAP administrations. It is important to note that during the second week of drug administration, one mouse in the D-gal + Vit E group became unexpectedly ill. Based on the veterinarian's recommendation, this animal was excluded from the study to prevent potential confounding effects on the results. As a result, the total number of mice in the D-gal + Vit E group was reduced to 9. At the end of treatment, all mice were sacrificed, and the sample collection was performed. The experimental design is illustrated in Figure 1.

### 2.2. Sample Collection

At the end of the treatment period, mice were anesthetized with an intraperitoneal injection of ketamine (100 mg/kg) [11]. After confirming deep anesthesia, a thoracotomy was performed to expose the heart. Blood was collected from the left ventricle using a sterile 1-mL syringe fitted with a 25G needle and placed in serum clot activator tubes. This terminal procedure allows for the collection of sufficient blood volume with minimal distress [25]. The blood was allowed to clot at room temperature, then centrifuged at 10, 000 × g for 10 min at 4°C to obtain serum, which was stored at −80°C until further analysis [26]. Upon concluding the experiment, half of the mice (5 animals per group) were culled to obtain fresh brains, while the remaining half (4-5 animals per group) were designated for another histopathological study. The required sample size was determined using G^∗^Power 3.1 [27] based on data from a previous study [14]. For fresh brain collection, mice were transcardially perfused with chilled phosphate buffer solution (PBS, pH 7.4) followed by rapid brain removal, dissection, and storage of the brain at −80°C until analysis, adhering to the established dissection protocol [28]. For histopathological and immunohistochemical (IHC) studies, mice were transcardially perfused with chilled PBS and subsequently with 10% neutral-buffered formalin (NBF) until rigor mortis set in. The brains and livers were then carefully removed and immersed in NBF for 48 h at 4°C prior to tissue processing.

### 2.3. Biochemical and Histopathological Assessment of Liver Function

The serum levels of three main liver enzymes, including aspartate aminotransferase (AST), alanine aminotransferase (ALT), and alkaline phosphatase (ALP), were measured in each sample by the Laboratory Unit, Research Institute for Health Sciences, Walailak University, Thailand. Hepatic histopathology was conducted using a standard protocol for H&E staining.

### 2.4. Malondialdehyde (MDA) Assay

Quantification of MDA contained in the brain tissues was performed using a commercial kit (cat no. MAK085, Sigma-Aldrich, St. Louis, MO, USA), and the protocols were in accordance with the manufacturer's instructions. Briefly, frozen frontal cortical and hippocampal tissues were lysed in 300-µL chilled MDA lysis buffer containing 3-µL butylated hydroxytoluene (BHT, 100×). The brain lysates were centrifuged for 10 min at 10, 000 × g, and the supernatants were kept. A series of MDA standard solutions was prepared to concentrations of 0.4, 0.8, 1.2, 1.6, and 2.0 nM. Subsequently, each sample and standard were mixed with 600-µL thiobarbituric acid (TBA) solution. The mixtures were boiled at 95°C for 30 min and then chilled in an ice bath for 10 min. To measure the optical density, 200 µL of the solution was pipetted into a 96-well plate in duplicate and read at 532 nm using a microplate reader. The concentration of MDA in the samples was determined by plotting against the curve of the standard, dividing by the sample volume (mL) added to the wells, and multiplying by the respective dilution factors. The amount of protein contained in the sample was quantified using the Bradford method [29], and the MDA content was reported as nmol/mg protein.

### 2.5. Neuronal Histopathological Study

Formalin-fixed brain tissues were processed and embedded in paraffin wax. Each paraffin-embedded block of the brain was sectioned coronally at a thickness of 5 μm and placed on a glass slide. Five animals were selected per group, except for the D-gal + Vit E group (*n* = 4). From each animal, tissue sections were collected at every eighth interval, and three sections were selected for analysis. After air-drying, the sections were stained with H&E using a standard protocol. All slides were scanned using a digital microscope and a slide scanner (M8 Precipoint, Fritz Müller, Germany). Cell counting in the frontal cortex (approximately 1.94 to 2.34 mm from bregma) [30] was performed inside a standard measuring frame (82,875 μm^2^) in five random fields from each section, while the number of neuronal cells in the whole CA1 area was counted for the hippocampus (approximately −1.90 to −2.10 mm from bregma) [30]. Cell counting in each brain was performed at a magnification of 400× using ViewPoint and virtual slide-viewing software. Neurons that exhibited unchanged histological features of the nucleus and cell membrane were classified as intact or surviving neurons [31]. Pathological changes were characterized by cellular shrinkage, chromatin condensation, pyknosis of the nucleus, and eosinophilia [32]. The number of intact neurons is represented as the number of cells per high-power field, and the number of damaged cells is reported as a percentage relative to the total number of cells. The neuronal cell counting was conducted by an experimenter who was blinded to the experimental conditions.

### 2.6. IHC Examination

Three representative slides from each brain containing the hippocampus and frontal cortex, consistent with those used in the histological study, were chosen to conduct the quantitative study. The images of five random fields within an area of 82,875 µm^2^ from the frontal cortex and the hippocampal CA1 regions (covering the stratum radiatum, pyramidal cell layer, and stratum oriens) were captured at a high magnification (400×) with AxioM1 light microscope (Carl Zeiss, Oberkochen, Germany) equipped with a digital camera (Axiocam, Carl Zeiss) connected to a PC monitor.

The quantitative immunoreactivity of TNF-α, IL-1β, IL-10, TGF-β, and NADPH Oxidase 4 (NOX4) in all captured images was monitored using ImageJ software (National Institutes of Health, Bethesda, MD, USA). Every photograph taken has a resolution of 1771 × 945 pixels. The original RGB color image was converted to 8-bit grayscale by subtracting the background. The DAB-stained pictures produced by color deconvolution were utilized to examine IHC staining. The brightness of each pixel in the DAB color image was represented by a value between 0 (black) and 255 (white). Using a threshold of 100, the total area of the image with moderate-to-strong IHC staining was established. The percentage of the image with moderate-to-strong staining was then calculated by dividing the values by the image's entire area [33]. The relative immunoreactivity (RI) ratio for each antibody was calculated as a percentage, with 100% corresponding to the control group. Quantitative analysis of immunoreactivity was conducted by an experimenter blinded to the experimental conditions.

### 2.7. Statistical Analysis

In this study, one-way analysis of variance (ANOVA) and Bonferroni's post hoc test were used to statistically evaluate all the data. All analyses were carried out utilizing GraphPad Prism 9.1 (GraphPad Software Inc., La Jolla, CA, USA). Statistical significance was set at *p* < 0.05, and the results are displayed as the mean ± SD.

## 3. Results

### 3.1. Liver Function Enzymes and Hepatic Histomorphology

The serum levels of the three main liver enzymes did not differ significantly among the experimental animals. The examination of hepatic morphology by H&E staining demonstrated no significant differences in terms of cell necrosis, binucleated hepatocytes, cell swelling, or inflammatory cell infiltration among the experimental groups (Supporting Figure 1).

### 3.2. Neuronal Histopathology

**Figure 2:**
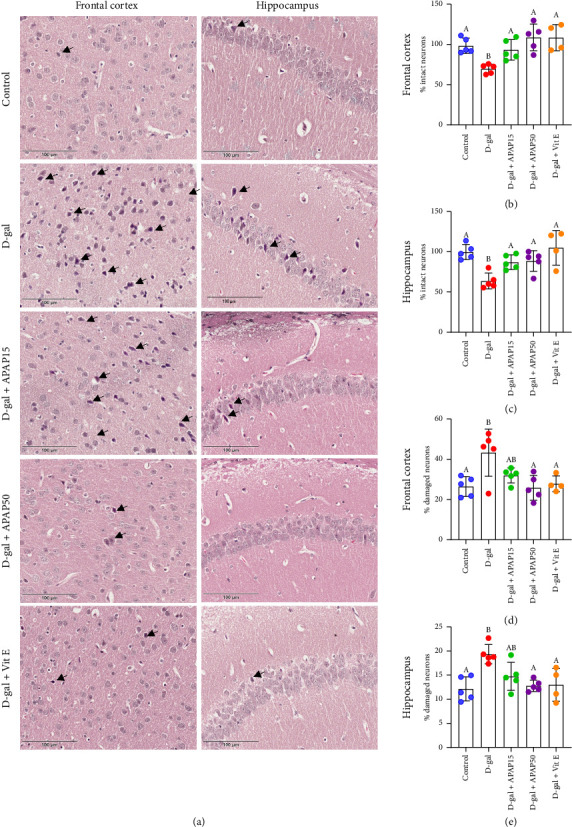
Effects of low-dose APAP treatment on the number of intact neurons and damaged neurons in the frontal cortex and hippocampus of D-gal-induced aging mice. (a) Representative histopathological micrographs of the frontal cortex and hippocampus across five groups: control, D-gal, D-gal + APAP15, D-gal + APAP50, and D-gal + Vit E. All sections were stained and imaged under identical conditions. Images were captured at 400× magnification. Scale bar = 100 μm. (b, c) Quantitative analysis of the number of intact neurons in the frontal cortex (b) and hippocampus (c). (d, e) Quantitative analysis of the number of damaged neurons in the frontal cortex (d) and hippocampus (e). Data are presented as mean ± SD (*n* = 4-5 mice per group). Statistical significance was determined using one-way ANOVA followed by Bonferroni's post hoc comparison. Histogram bars that do not share a common letter are significantly different at *p* < 0.05. Abbreviations: APAP15, low-dose paracetamol at 15 mg/kg; APAP50, low-dose paracetamol at 50 mg/kg; Vit E, vitamin E.

H&E staining was used to illustrate morphological changes in frontal cortical and hippocampal CA1 neurons (Figure 2(a)). Few injuries were detected in the control group. There was no edema surrounding the neurons in the visual field, and the neurons were all clear and undamaged. However, a significant proportion of neurons in the D-gal group showed notable degenerative alterations, such as eosinophilia, pericellular edema, loss of integrity, cell shrinkage, and nucleus pyknosis.

The severity of neuronal degeneration in the group receiving D-gal injection was reduced by the injection of low-dose APAP (15 and 50 mg/kg) or Vit E. Figure 2(b) shows a quantitative analysis of cell counting in the frontal cortex, revealing a significant 30.10% decrease in the number of intact neurons in the D-gal group compared to the control group (69.90 ± 5.82% vs. 100.00 ± 9.51%, *p* < 0.05). Compared to the D-gal group, a significant increase in intact neurons was observed in the D-gal + APAP15, D-gal + APAP50, and D-gal + Vit E groups, with increases of 23.74%, 39.16%, and 38.85%, respectively (93.64 ± 12.85, 109.10 ± 16.67, and 108.80 ± 16.63% vs. 69.90 ± 5.82%; *p* < 0.05).

Results obtained from cell counting in the hippocampus are illustrated in Figure 2(c). A significant 36.12% decrease in the number of intact neurons was observed in the D-gal group compared to the control group (63.88 ± 9.93% vs. 100.00 ± 9.32%, *p* < 0.05). However, we observed a significant increase in the number of intact neurons in the D-gal + APAP15, D-gal + APAP50, and D-gal + Vit E groups compared with the D-gal group, with increases of 23.47%, 24.86%, and 41.46%, respectively (87.35 ± 9.07, 88.74 ± 12.97, and 105.30 ± 21.66% vs. 63.88 ± 9.93%, *p* < 0.05).

The percentage of damaged neurons in the frontal cortex of the D-gal group was significantly increased (16.86%) compared with the control group (43.40 ± 11.78% vs. 26.54 ± 4.93%, *p* < 0.05), as shown in Figure 2(d). There was no significant difference in percentage of damaged neurons in the D-gal + APAP15 group compared with the D-gal group. However, significant decreases in the percentage of damaged neurons were observed in the D-gal + APAP50 group (17.50%) and D-gal + Vit E group (15.55%) compared to the D-gal group (25.90 ± 6.18 and 27.85 ± 3.93% vs. 43.40 ± 11.78%, *p* < 0.05).

The percentage of damaged neurons in the hippocampus among all animal groups is illustrated in Figure 2(e), revealing a significant increase in the D-gal group (7.20%) compared with the control group (19.40 ± 2.00% vs. 12.20 ± 2.49%, *p* < 0.05). No significant difference in the percentage of damaged neurons was detected in the D-gal + APAP15 group compared to the D-gal group. However, significant decreases were observed in the D-gal + APAP50 group (6.58%) and D-gal + Vit E group (6.37%) compared to the D-gal group (12.82 ± 1.18 and 13.03 ± 3.40% vs. 19.40 ± 2.00%, *p* < 0.05).

### 3.3. MDA Levels

**Figure 3:**
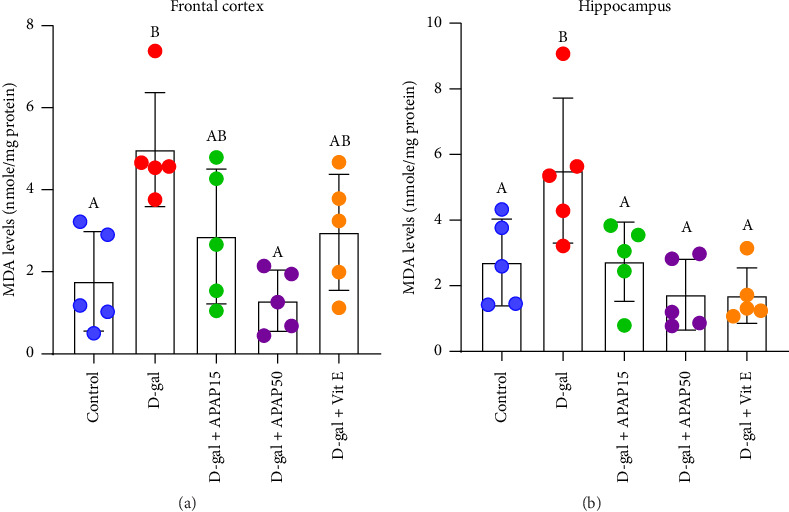
Effects of low-dose APAP treatment on the MDA levels in the frontal cortex and hippocampus of D-gal-induced aging mice. (a, b) The alteration of MDA levels in the frontal cortex (a) and hippocampus (b) across five groups: control, D-gal, D-gal + APAP15, D-gal + APAP50, and D-gal + Vit E. Data are presented as mean ± SD (*n* = 5 mice per group). Statistical significance was determined using one-way ANOVA followed by Bonferroni's post hoc comparison. Histogram bars that do not share a common letter are significantly different at *p* < 0.05. Abbreviations: APAP15, low-dose paracetamol at 15 mg/kg; APAP50, low-dose paracetamol at 50 mg/kg; Vit E, vitamin E; MDA, malondialdehyde.

The MDA levels in the experimental groups varied significantly. In the frontal cortex, the MDA level was significantly increased in the D-gal group compared to that in the control group (5.00 ± 1.39 vs. 1.77 ± 1.22 nmol/mg protein, *p* < 0.05), as shown in Figure 3(a). Neither the D-gal + APAP15 nor the D-gal + Vit E group exhibited a significant difference in MDA levels compared with the D-gal group (*p* > 0.05). However, a significant decrease in the MDA level was demonstrated in the D-gal + APAP50 group compared to the D-gal group (1.29 ± 0.75 vs. 5.00 ± 1.39 nmol/mg protein, *p* < 0.05). In the hippocampus (Figure 3(b)), a significant increase in the MDA level was observed in the D-gal group when compared with the control group (5.53 ± 2.21 vs. 2.72 ± 1.32 nmol/mg protein, *p* < 0.05). However, MDA levels in the D-gal + APAP15, D-gal + APAP50, and D-gal + Vit E groups were significantly decreased compared to the D-gal group (2.74 ± 1.21, 1.73 ± 1.08, and 1.70 ± 0.84 vs. 5.53 ± 2.21 nmol/mg protein, respectively; *p* < 0.05).

### 3.4. Immunohistochemistry (IHC) Analysis

**Figure 4:**
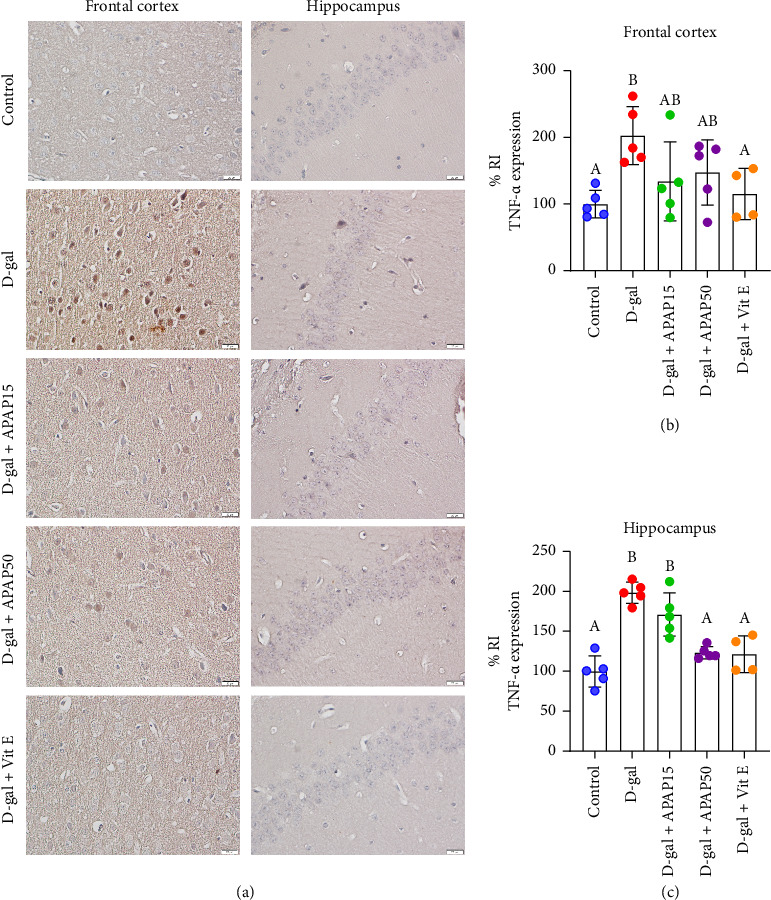
Effects of low-dose APAP treatment on TNF-α expression in the frontal cortex and hippocampus of D-gal-induced aging mice. (a) Representative immunohistochemical images showing TNF-α immunoreactivity in the frontal cortex and hippocampus across five groups: control, D-gal, D-gal + APAP15, D-gal + APAP50, and D-gal + Vit E. All sections were stained and imaged under identical conditions. Images were captured at 400× magnification. Scale bar = 20 μm. (b, c) Quantitative analysis of TNF-α relative immunoreactivity (% RI) in the frontal cortex (b) and hippocampus (c). Data are presented as mean ± SD (*n* = 4-5 mice per group). Statistical significance was determined using one-way ANOVA followed by Bonferroni's post hoc comparison. Histogram bars that do not share a common letter are significantly different at *p* < 0.05. Abbreviations: APAP15, low-dose paracetamol at 15 mg/kg; APAP50, low-dose paracetamol at 50 mg/kg; Vit E, vitamin E; RI, relative immunoreactivity; TNF-α, tumor necrosis factor-α.

IHC analysis revealed that TNF-α protein expression in the frontal cortex was significantly elevated by 103.2% in the D-gal group compared to the control group (203.2 ± 43.59% vs. 100.0 ± 20.71%, *p* < 0.05; Figures 4(a) and 4(b)). Compared to the D-gal group, TNF-α protein expression was not significantly altered in the D-gal + APAP15 and D-gal + APAP50 groups. However, a marked decrease in TNF-α protein (87.87%) was observed in the D-gal + Vit E group (115.4 ± 38.48% vs. 203.2 ± 43.59%, *p* < 0.05). In the hippocampal CA1, TNF-α protein expression was significantly increased by approximately 99% in the D-gal group compared with the control group (199.0 ± 13.28% vs. 100.0 ± 19.64%, *p* < 0.05, Figures 4(a) and 4(c)). No significant difference in TNF-α protein levels was observed between the D-gal + APAP15 and the D-gal groups. However, a 75.30% decrease in TNF-α immunoreactivity was detected in the D-gal + APAP50 group (123.7 ± 7.72% vs. 199.0 ± 13.28%, *p* < 0.05). Likewise, the D-gal + Vit E group exhibited a significant reduction in TNF-α immunoreactivity (77.14%) compared to the D-gal group (121.9 ± 23.10% vs. 199.0 ± 13.28%, *p* < 0.05).

**Figure 5:**
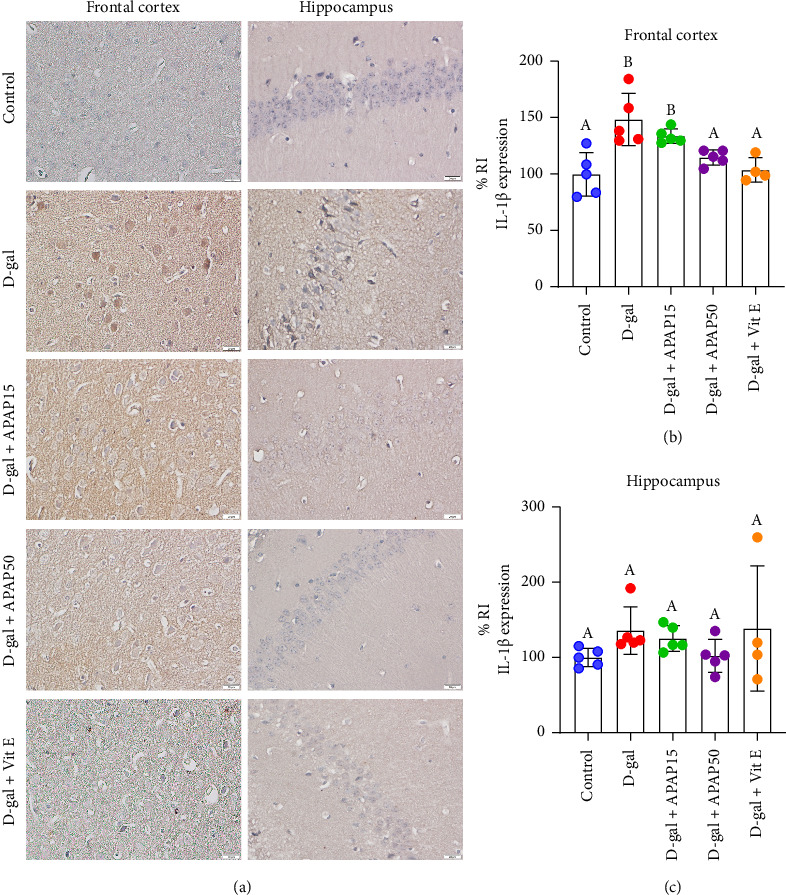
Effects of low-dose APAP treatment on IL-1β expression in the frontal cortex and hippocampus of D-gal-induced aging mice. (a) Representative immunohistochemical images showing IL-1β immunoreactivity in the frontal cortex and hippocampus across five groups: control, D-gal, D-gal + APAP15, D-gal + APAP50, and D-gal + Vit E. All sections were stained and imaged under identical conditions. Images were captured at 400× magnification. Scale bar = 20 μm. (b, c) Quantitative analysis of IL-1β relative immunoreactivity (% RI) in the frontal cortex (b) and hippocampus (c). Data are presented as mean ± SD (*n* = 4–5 mice per group). Statistical significance was determined using one-way ANOVA followed by Bonferroni's post hoc comparison. Histogram bars that do not share a common letter are significantly different at *p* < 0.05. Abbreviations: APAP15, low-dose paracetamol at 15 mg/kg; APAP50, low-dose paracetamol at 50 mg/kg; Vit E, vitamin E; RI, relative immunoreactivity; IL-1β, interleukin-1β.

The immunoreactivity of IL-1β in both the frontal cortex and hippocampal CA1 is displayed in Figure 5(a). Quantitative analysis in the frontal cortex revealed a significant increase in the expression of IL-1β in the D-gal group (48.80%) compared to the control (148.8 ± 23.23% vs. 100.0 ± 19.41%, *p* < 0.05, Figure 5(b)). IL-1β protein expression in the D-gal + APAP15 group did not significantly differ from that observed in the D-gal group (*p* > 0.05). However, the D-gal + APAP50 group showed a significant decrease in IL-1β protein expression, with a 33.76% decrease of IL-1β immunoreactivity compared with the D-gal mice (115.0 ± 6.72% vs. 148.8 ± 23.23%, *p* < 0.05). Furthermore, we detected a significant decrease in IL-1β protein expression in the D-gal + Vit E group, with a 44.85% decrease of IL-1β immunoreactivity compared to the D-gal group (103.9 ± 10.84% vs. 148.8 ± 23.23%, *p* < 0.05; Figure 5(c)). In an observation of the hippocampal CA1, significant differences in IL-1β immunoreactivity were not detected among the five experimental groups (*p* > 0.05; Figure 5(c)).

**Figure 6:**
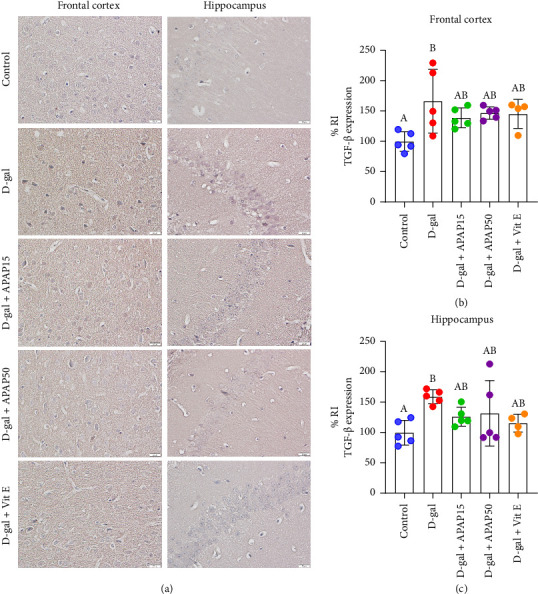
Effects of low-dose APAP treatment on TGF-β expression in the frontal cortex and hippocampus of D-gal-induced aging mice. (a) Representative immunohistochemical images showing TGF-β immunoreactivity in the frontal cortex and hippocampus across five groups: control, D-gal, D-gal + APAP15, D-gal + APAP50, and D-gal + Vit E. All sections were stained and imaged under identical conditions. Images were captured at 400× magnification. Scale bar = 20 μm. (b, c) Quantitative analysis of TGF-β relative immunoreactivity (% RI) in the frontal cortex (b) and hippocampus (c). Data are presented as mean ± SD (*n* = 4-5 mice per group). Statistical significance was determined using one-way ANOVA followed by Bonferroni's post hoc comparison. Histogram bars that do not share a common letter are significantly different at *p* < 0.05. Abbreviations: APAP15, low-dose paracetamol at 15 mg/kg; APAP50, low-dose paracetamol at 50 mg/kg; Vit E, vitamin E; RI, relative immunoreactivity; TGF-β, transforming growth factor-β.

TGF-β immunoreactivity in the frontal cortex and hippocampal CA1 is shown in Figure 6(a). Observation in the frontal cortex revealed a significant increase in TGF-β protein expression in the D-gal group, with an approximately 66% increase in TGF-β immunoreactivity compared to the control group (166.7 ± 52.63% vs. 100.0 ± 16.28%, *p* < 0.05, Figure 6(b)). However, there were no significant changes in TGF-β immunoreactivity in the D-gal + APAP15, D-gal + APAP50, or D-gal + Vit E groups compared to the D-gal group (*p* > 0.05). The findings from the hippocampal CA1 region revealed a significant increase in TGF-β protein in the D-gal group (59.29%) compared to the control group (159.29 ± 11.61% vs. 100.0 ± 9.14%, *p* < 0.05, Figure 6(c)). Nevertheless, we could not detect a statistically significant difference in the D-gal mice treated with low-dose APAP (15 or 50 mg/kg), or Vit E (*p* > 0.05).

**Figure 7:**
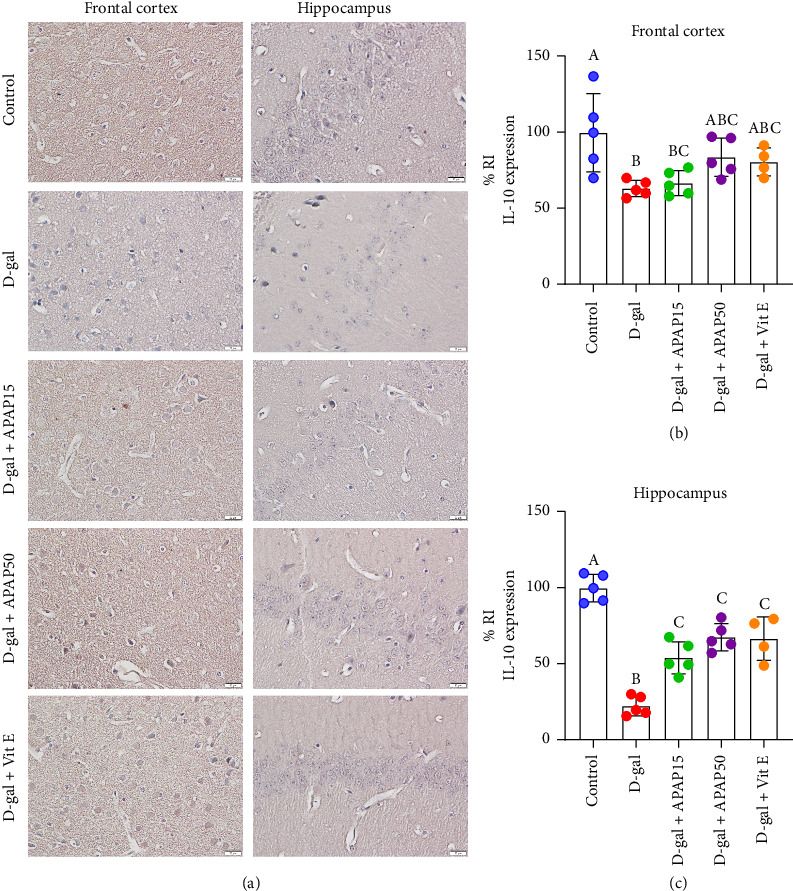
Effects of low-dose APAP treatment on IL-10 expression in the frontal cortex and hippocampus of D-gal-induced aging mice. (a) Representative immunohistochemical images showing NOX4 immunoreactivity in the frontal cortex and hippocampus across five groups: control, D-gal, D-gal + APAP15, D-gal + APAP50, and D-gal + Vit E. All sections were stained and imaged under identical conditions. Images were captured at 400× magnification. Scale bar = 20 μm. (b, c) Quantitative analysis of IL-10 relative immunoreactivity (% RI) in the frontal cortex (b) and hippocampus (c). Data are presented as mean ± SD (*n* = 4-5 mice per group). Statistical significance was determined using one-way ANOVA followed by Bonferroni's post hoc comparison. Histogram bars that do not share a common letter are significantly different at *p* < 0.05. Abbreviations: APAP15, low-dose paracetamol at 15 mg/kg; APAP50, low-dose paracetamol at 50 mg/kg; Vit E, vitamin E; RI, relative immunoreactivity; IL-10, interleukin-10.

The immunoreactivity of IL-10 in the frontal cortex and hippocampal CA1 is shown in Figure 7(a). In the frontal cortex, statistical analysis revealed a significant decrease in IL-10 protein expression in the D-gal group, with a 36% reduction in IL-10 immunoreactivity compared to the control group (63.16 ± 5.35% vs. 100.0 ± 25.85%, *p* < 0.05, Figure 7(b)). We could not detect a significant difference in IL-10 protein expression in the D-gal + APAP15, D-gal + APAP50, or D-gal + Vit E groups when compared with the D-gal group (*p* > 0.05). Statistical analysis in the hippocampal CA1 (Figure 7(c)) demonstrated that the IL-10 protein was significantly decreased in the D-gal group, with a 77.81% decrease in IL-10 immunoreactivity compared to the control group (22.19 ± 6.47% vs. 100.00 ± 9.09%, *p* < 0.05). However, significant increases in the IL-10 protein expression were observed in the D-gal + APAP15 (31.81%), D-gal + APAP50 (45.39%), and D-gal + Vit E (44.49%) groups compared with the D-gal group (53.99 ± 10.64, 67.57 ± 9.04, and 66.68 ± 14.30% vs. 22.19 ± 6.47%, respectively, *p* < 0.05).

**Figure 8:**
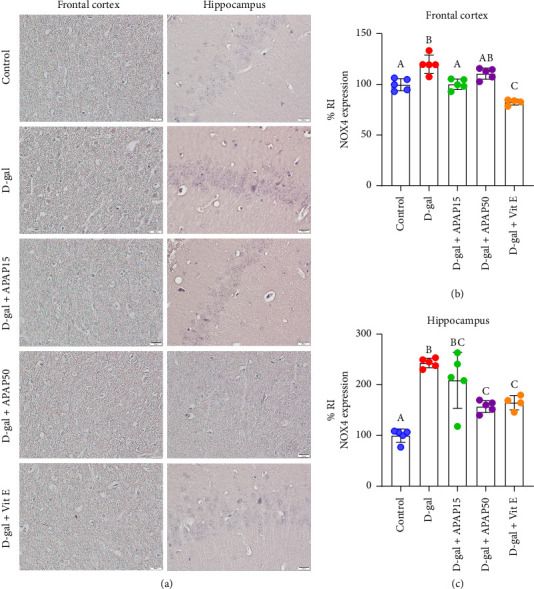
Effects of low-dose APAP treatment on NOX4 expression in the frontal cortex and hippocampus of D-gal-induced aging mice. (a) Representative immunohistochemical images showing NOX4 immunoreactivity in the frontal cortex and hippocampus across five groups: control, D-gal, D-gal + APAP15, D-gal + APAP50, and D-gal + Vit E. All sections were stained and imaged under identical conditions. Images were captured at 400× magnification. Scale bar = 20 μm. (b, c) Quantitative analysis of NOX4 relative immunoreactivity (% RI) in the frontal cortex (b) and hippocampus (c). Data are presented as mean ± SD (*n* = 4-5 mice per group). Statistical significance was determined using one-way ANOVA followed by Bonferroni's post hoc comparison. Histogram bars that do not share a common letter are significantly different at *p* < 0.05. Abbreviations: APAP15, low-dose paracetamol at 15 mg/kg; APAP50, low-dose paracetamol at 50 mg/kg; Vit E, vitamin E; RI, relative immunoreactivity; NOX4, NADPH oxidase 4.

The photograph of the NOX4 immunoreactivity in both the frontal cortex and hippocampal CA1 is provided in Figure 8(a). Figure 8(b) shows a statistical analysis of NOX4 immunoreactivity in the frontal cortex, revealing a significant increase in NOX4 protein expression in the D-gal mice, with a 20.23% increase in NOX4 immunoreactivity compared to the control group (120.2 ± 9.19% vs. 100.0 ± 5.93%, *p* < 0.05). However, compared to the D-gal group, the protein expression of NOX4 was significantly decreased in the D-gal + APAP15 (19.76%) and D-gal + Vit E (37.54%) groups (100.5 ± 5.15 and 82.6 ± 2.79% vs. 120.2 ± 9.19%, *p* < 0.05). In this study, NOX protein expression in the D-gal + APAP50 group did not differ significantly from that in the D-gal group. A quantitative analysis of NOX4 immunoreactivity in the hippocampal CA1 is shown in Figure 8(c). A significant increase in NOX protein expression was observed in the D-gal group, with a 144.10% increase in NOX4 immunoreactivity compared to the control group (244.1 ± 9.34% vs. 100.0 ± 13.16%, *p* < 0.05). Compared to the D-gal group, there was no significant difference in NOX protein expression in the D-gal + APAP15 group (*p* < 0.05). However, the NOX4 protein was significantly decreased in the D-gal + APAP50 (86.38%) and D-gal + Vit E (78.76%) groups compared to the D-gal group (157.7 ± 11.70 and 165.3 ± 14.17% vs. 244.1 ± 9.34%, *p* < 0.05).

## 4. Discussion

The D-gal-induced aging mouse model employed in this study is well established for mimicking key aspects of natural aging, particularly increased oxidative stress and chronic inflammation [9, 11, 12]. Prolonged D-gal administration enhances the production of ROS and impairs antioxidant defenses, resulting in oxidative damage. Concurrently, it activates neuroinflammatory pathways, including the upregulation of cytokines such as TNF-α and IL-1β. These alterations closely resemble pathological features observed in age-related neurodegenerative disorders [5, 8]. In our present study, the D-gal-treated mice exhibited elevated oxidative stress, marked by increased MDA and NOX4 levels, along with heightened neuroinflammation, as shown by upregulated TNF-α, IL-1β, and TGF-β, and reduced IL-10. These findings further support the relevance of the model for investigating mechanisms underlying age-related neurodegeneration.

Over the past decade, several studies have reported the neuroprotective effects of low-dose APAP treatment in various experimental models. Our previous work demonstrated that 6-week treatment with low-dose APAP (15 and 50 mg/kg) enhanced cognitive function, as evidenced by improved performance in NOR and MWM tests in the D-gal-induced aging mice [19]. However, the mechanisms underlying these beneficial effects remain unclear. In the present study, we expand upon our previous findings by investigating whether the antioxidant and anti-inflammatory properties of low-dose APAP contribute to its ability to attenuate cognitive decline in the same cohort of D-gal-induced aging mice. The results showed that low-dose APAP treatment (15 and 50 mg/kg) attenuated neuronal loss in the key brain regions of the D-gal aging mouse model. In addition, low-dose APAP improved the oxidative stress status and inflammatory mediator profiles in the aging brain. Collectively, the protective effects of APAP at the low doses employed in this study were similar to those exhibited by Vit E administration.

APAP is an over-the-counter medication because of its wide safety margin. However, large doses of this drug are strongly linked to hepatotoxicity [34]. The results in this study demonstrated that neither low-dose APAP (15 or 50 mg/kg) nor D-gal administration induced hepatotoxicity, as evidenced by the absence of significant changes in the liver enzyme levels (AST, ALT, and ALP), and hepatic histology across all the experimental groups (see Supporting Figure 1). These results suggest that chronic low-dose APAP treatment causes no harm to the liver, and the neuropathological manifestations identified in this study were not induced by hepatotoxicity.

The frontal cortex and hippocampus are especially vulnerable to age-related changes and neurodegenerative processes, impacting various forms of plasticity, learning, and memory [35, 36]. The CA1 sector of the hippocampus is one of the first brain regions to display pathology and neuronal loss in AD [37]. Hence, these are the key regions of interest in studies focusing on aging and neurodegeneration. In this study, mice chronically treated with D-gal alone showed a decrease in intact neurons and an increase in neuronal damage in both the frontal cortex and the hippocampus. These results are in accordance with those of several studies that have shown an increase in senescent cells and apoptosis in the brain following D-gal induction [21, 38]. On the other hand, D-gal mice given low-dose APAP (15 and 50 mg/kg), or Vit E showed an increase in the number of intact neurons. The treatment with 50 mg/kg APAP and Vit E markedly decreased neuronal damage. These results suggest that low-dose APAP and Vit E treatments exert antiaging effects in the D-gal-induced accelerated aging brain. The neuroprotective effects of low-dose APAP against neuronal cell damage may also account for the improved performance in NOR and MWM tasks observed in D-gal-induced aging mice in a previous study [19].

The rapid accumulation of free radicals that accelerates the aging of tissues and organs is the most advantageous aspect of the D-gal aging model [10]. Several brain regions, including the auditory cortex, hippocampus, ventral cochlear nucleus, and cerebral cortex, could be affected by D-gal-induced oxidative stress and mitochondrial dysfunction [39–41]. NOX4 is a member of the NOX family of oxidases, which is the major source of ROS production in mammals [42]. At the physiological level, it typically exists in the brain and has a role in the elimination of toxic chemicals from cells and in cell signal transduction. However, under pathological conditions, overexpression of NOX4 is strongly associated with oxidative stress and is mediated by senescent cells [43]. In our study, elevated MDA levels were accompanied by increased NOX4 expression in both the frontal cortex and hippocampus of the D-gal-treated mice. These suggest that the upregulation of NOX4 observed in the D-gal-treated mice may contribute to the elevated oxidative stress. Overproduction of ROS resulting from increased NOX4 levels may result in augmented MDA content, which can contribute to cytotoxicity by polymerizing and cross-linking large molecules including proteins and nucleic acids.

After low-dose APAP and Vit E interventions, the MDA content in D-gal-aged rats decreased to varying degrees, especially in the hippocampal CA1 region. These results indicate that low-dose APAP can reduce damage caused by peroxidation, helping to moderate the degree of aging. Moreover, low-dose APAP and Vit E decreased NOX4 levels in the brain areas of interest. The potential antioxidant impact of Vit E and its ability to lower the risk of memory decline in AD and aging have been recognized [24, 44]. Numerous genes implicated in growth factors, hormone metabolism, neurotransmission, apoptosis, and amyloid-β (Aβ) metabolism are associated with the development of AD, all of which are modulated by Vit E [45]. At the same time, the antioxidant activity of APAP against neurotoxin-induced cytotoxicity has been reported in both in vitro and in vivo studies [17, 46, 47]. Our findings are consistent with those of a previous study, which demonstrated a reduction in MDA levels in colchicine-induced amnesic rats following administration of APAP at a dose of 15.1 mg/kg [16]. Moreover, we observed that APAP at 15 mg/kg showed greater potential for reducing NOX4 levels in the frontal cortex, while a dose of 50 mg/kg was more effective in the hippocampus. These findings may reflect the distinct oxidative environments and regional sensitivities in the brain. The frontal cortex may be more responsive to low-dose antioxidant action, whereas the hippocampus, which is more metabolically active and susceptible to oxidative stress, may require a higher dose to trigger protective mechanisms [48]. Furthermore, APAP exhibits dose-dependent neuroprotective effects, which may involve antioxidant and anti-inflammatory pathways at lower doses [49]. The apparent biphasic response may be explained by the concept of hormesis, where low doses of a compound elicit beneficial effects, but higher doses are less effective or even counterproductive [50]. Lastly, the regulation of NOX4 differs across brain regions, which may underlie these variable responses [51]. Further investigation into the metabolism of varying doses across different brain areas may provide valuable insights. Regarding the results obtained from this study, the neuroprotective effects of low-dose APAP treatment in the D-gal aging model may be attributed, at least in part, to its antioxidant properties.

Apart from oxidative stress, persistent immune responses and chronic inflammation are considered to play major roles in age-related diseases [52]. In neurodegenerative disorders, such as AD and PD, elevated levels of TNF-α, IL-1β, and TGF-β have been detected, highlighting the significant influence of these cytokines in the onset and progression of these disorders [53, 54]. In our experiments, we observed increased levels of TNF-α, IL-1β, and TGF-β in both the frontal cortex and hippocampus of D-gal-treated mice, suggesting neuroinflammation. Additionally, there was a decrease in IL-10 expression in both brain regions. IL-10 plays a critical role in immune response regulation and inflammation suppression. Although IL-10 is generally acknowledged for its protective attributes owing to its anti-inflammatory characteristics, its involvement in neurodegenerative conditions such as AD and PD is complex and multifaceted [55]. TGF-β is a pleiotropic cytokine, and disrupting its signaling can decrease the ability of microglia to respond to damage [56]. However, during aging, elevated TGF-β levels have been observed in the hippocampus and hypothalamus, among other brain regions [57]. The increased levels of TNF-α, IL-1β, and TGF-β in D-gal-treated mice may exacerbate neuroinflammation and contribute to neurodegeneration. Several studies have liked neuroinflammation to oxidative stress. Upregulation of cytokines can activate microglia and astrocytes to release ROS and reactive nitrogen species (RNS) as part of their immune response [52]. TNF-α and IL-1β can activate various proinflammatory signaling pathways, including nuclear factor-kappa B (NF-κB) and mitogen-activated protein kinases (MAPKs) which can further promote the expression of inflammatory mediators and contribute to oxidative stress by inducing the expression of ROS-generating enzymes, such as NOX4, and inhibiting antioxidant defenses [58]. Oxidative stress, in turn, has the capacity to induce the expression of TNF-α, IL-1β, and TGF-β through several pathways, including activating NF-κB and activator protein-1 (AP-1) [59], and modulating MAPKs, such as p38 MAPK and c-Jun N-terminal kinase (JNK), which are known to regulate the expression of proinflammatory cytokines [60]. The results obtained from our study suggest that D-gal injection for 6 weeks induces increased oxidative stress and neuroinflammation, which can lead to cellular damage and ultimately contribute to neuronal degeneration.

In this study, administration of 50 mg/kg of APAP significantly reduced TNF-α levels in the hippocampus and IL-1β levels in the frontal cortex. However, Vit E exhibited even greater efficacy in lowering these proinflammatory cytokines. Notably, both Vit E and low-dose APAP, particularly at 50 mg/kg, also restored IL-10 levels in the examined brain regions. These findings indicate that low-dose APAP exerts neuroprotective effects in the D-gal-induced aging brain model through its anti-inflammatory properties. Nevertheless, no significant changes in TGF-β levels were observed following treatment with either low-dose APAP or Vit E. The persistence of TGF-β expression may reflect age-related dysregulation of multiple signaling pathways, such as interferon gamma (IFN-γ) and NF-κB signaling [61]. While our results clearly demonstrate that low-dose APAP reduces oxidative stress and modulates key inflammatory cytokines, the underlying molecular mechanisms remain to be elucidated. It is plausible that APAP exerts these effects through modulation of intracellular signaling pathways such as nuclear factor Erythroid 2–related Factor 2 (Nrf2), which governs antioxidant enzyme expression, or the NF-κB pathway, which regulates the production of inflammatory cytokines [59]. Additionally, MAPK cascades may also play a role in mediating these effects [60].

Alternatively, research suggests that *N*-arachidonoylphenolamine (AM404), a metabolite of APAP, has the ability to modulate the cannabinoid (CB) system, hence mediating analgesic and anxiolytic-like effects [62]. In general, the activation of CB receptors, including CB1 and CB2, which are expressed throughout the CNS and peripheral nervous system (PNS), enhances cell proliferation and neurogenesis [63]. CB can also act as a neuromodulator and immunomodulatory agent to promote the physiological functions of both the CNS and PNS [64]. In a recent study, it was demonstrated that CB can delay the aging process of the brain and provide neuroprotective antioxidant effects through its metal ion chelating properties [65]. Moreover, CB may indirectly regulate ROS levels by controlling the glial activity in astrocytes and microglia [63]. During aging, the CB system may be impaired as many hormones and metabolic pathways' decline. Therefore, low-dose APAP may reduce oxidative stress and neuroinflammation in D-gal-aged mice through the interaction between the metabolite AM404 and the CB system, contributing to improved metabolic and immune regulation in the aging brain. Overall, our findings suggest that low-dose APAP modulates the release of inflammatory mediators, including TNF-α, IL-1β, and IL-10, but does not influence TGF-β levels in this aging model.

It should be noted that this study has limitations. First, it did not investigate specific signaling molecules (e.g., MAPK, NF-kB, and Nrf2) involved in the antioxidant and anti-inflammatory effects of low-dose APAP treatment. Incorporating these molecular markers in future studies could help clarify the underlying mechanisms. Second, there is a lack of evidence explaining the fluctuations of proinflammatory mediators across different brain regions following low-dose APAP exposure. The differential effects observed in the frontal cortex and hippocampus may be attributed to variations in blood flow or compound distribution. Future investigations into APAP distribution could help elucidate its region-specific effects.

## 5. Conclusion

Our in vivo study demonstrated that low-dose APAP treatment mitigated neuronal pathology in a mouse model of D-gal-induced aging. The observed neuroprotective effects may be associated with the antioxidant and anti-inflammatory properties of APAP. Although the molecular mechanisms of APAP are traditionally associated with hepatotoxicity at high doses, our findings highlight the potential therapeutic benefits of low-dose administration within a safe range. These results contribute to a growing body of evidence suggesting that low-dose APAP may serve as a promising prophylactic or adjunctive intervention for mitigating age-related neurodegenerative changes linked to oxidative stress and neuroinflammation. Nevertheless, further investigations, including well-designed clinical trials, are warranted to validate the translational relevance of these findings to human health.

## Data Availability

The data that support the findings of this study are available from the corresponding author upon reasonable request.

## Nomenclature

AD: Alzheimer's disease
ALP: Alkaline phosphatase
ALT: Alanine aminotransferase
AM404: *N*-arachidonoylphenolamine
ANOVA: Analysis of variance
APAP: Paracetamol
AST: Aspartate aminotransferase
Aβ: β-amyloid
BDNF: Brain-derived neurotrophic factor
BHT: Butylated hydroxytoluene
CAT: Catalase
CB: Cannabinoid
COX3: Cyclooxygenase-3
DAB: Diaminobenzidine
GSH: Glutathione peroxidase
GSH: Glutathione
H&E: Hematoxylin and eosin
IFN-γ: Interferon gamma
IHC: Immunohistochemistry
IL-10: Interleukin-10
IL-1β: Interleukin-1β
JNK: c-Jun N-terminal kinase
MAPKs: Mitogen-activated protein kinases
MDA: Malondialdehyde
NBF: Neutral-buffered formalin
NF-κB: Nuclear factor-kappa B
NOX4: NADPH Oxidase 4
PBS: Phosphate buffer solution
PD: Parkinson's disease
D-gal: D-galactose
(ROS: Reactive oxygen species
SOD: Superoxide dismutase
TGF-β: Transforming growth factor-β
TNF-α: Tumor necrosis factor-α
TrkB: Tyrosine kinase B
Vit E: Vitamin E

## Supporting Information

Supporting Figure 1: Effects of low-dose APAP treatment on the liver function in the D-gal-induced aging mouse model.
