# Supplementary material for: Low-Dose Paracetamol Treatment Protects Neuronal Oxidative Stress and Neuroinflammation in D-Galactose-Induced Accelerated Aging Model
Source: Scientifica (Cairo). 2025 Sep 18;2025:5559483. doi: 10.1155/sci5/5559483 (PMC12463536; doi:10.1155/sci5/5559483)
Supplement: Supporting Information — Additional supporting information can be found online in the Supporting Information section. [file 5559483.f1.pptx]

## Slide 1
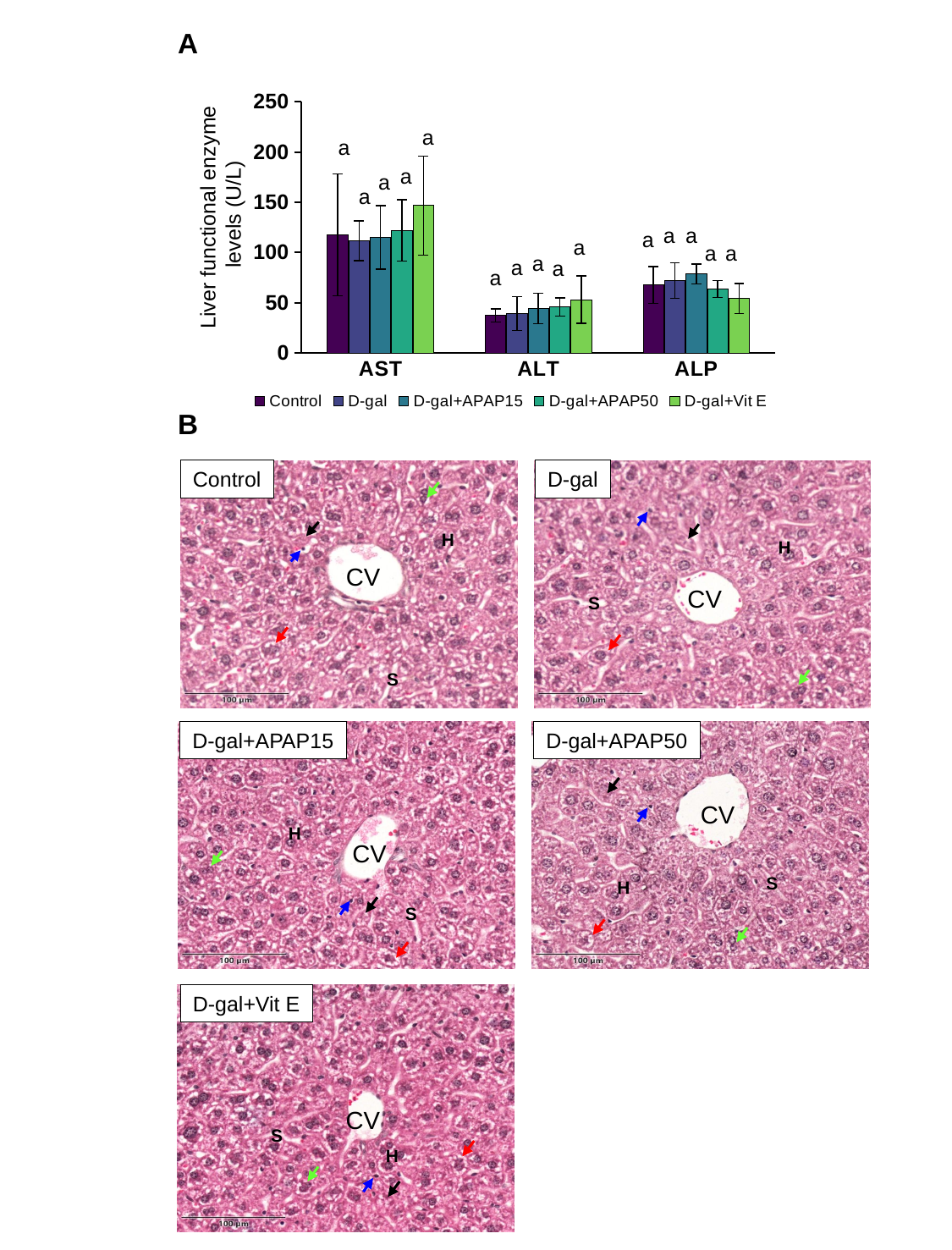

A
### Chart
| Category | Control | D-gal | D-gal+APAP15 | D-gal+APAP50 | D-gal+Vit E |
|---|---|---|---|---|---|
| AST | 117.5 | 111.8 | 115.0 | 122.0 | 146.7 |
| ALT | 37.2 | 39.4 | 44.4 | 45.75 | 53.0 |
| ALP | 67.78 | 72.23 | 78.54 | 63.6 | 54.27 |a
a
a
a
a
Liver functional enzyme
levels (U/L)
a
a
a
a
a
a
a
a
a
a
B
Control
D-gal
H
H
CV
CV
S
S
D-gal+APAP15
D-gal+APAP50
CV
H
CV
S
H
S
D-gal+Vit E
CV
S
H
